# The TWEAK/Fn14/CD163 axis—implications for metabolic disease

**DOI:** 10.1007/s11154-021-09688-4

**Published:** 2021-09-20

**Authors:** Wiktoria Ratajczak, Sarah D Atkinson, Catriona Kelly

**Affiliations:** Northern Ireland Centre for Stratified Medicine, School of Biomedical Sciences, Ulster University, Altnagelvin Hospital Campus, C-TRIC Building Glenshane Road, Derry/Londonderry, Northern Ireland UK

**Keywords:** TWEAK, FN14, CD163, Diabetes, Cardiovascular Disease, Metabolic Disease

## Abstract

TWEAK (tumor necrosis factor-like weak inducer of apoptosis) is a member of the TNF superfamily that controls a multitude of cellular events including proliferation, migration, differentiation, apoptosis, angiogenesis, and inflammation. TWEAK control of these events is via an expanding list of intracellular signalling pathways which include NF-κB, ERK/MAPK, Notch, EGFR and AP-1. Two receptors have been identified for TWEAK – Fn14, which targets the membrane bound form of TWEAK, and CD163, which scavenges the soluble form of TWEAK. TWEAK appears to elicit specific events based on the receptor to which it binds, tissue type in which it is expressed, specific extrinsic conditions, and the presence of other cytokines. TWEAK signalling is protective in healthy tissues, but in chronic inflammatory states become detrimental to the tissue. Consistent data show a role for the TWEAK/FN14/CD163 axis in metabolic disease, chronic autoimmune diseases, and acute ischaemic stroke. Low circulating concentrations of soluble TWEAK are predictive of poor cardiovascular outcomes in those with and without diabetes. This review details the current understanding of the TWEAK/Fn14/CD163 axis as one of the chief regulators of immune signalling and its cell-specific role in metabolic disease development and progression.

## Introduction

Members of the tumor necrosis factor (TNF) superfamily of cytokines are typically expressed as type 2 transmembrane proteins with homologous TNF domains. They are directly involved in immune responses and inflammation, in addition to proliferation, differentiation, apoptosis, and embryogenesis, and have consequently received attention as potential drug targets in recent years [1, 2].

TWEAK (TNFSF12) and its known receptor Fn14 (TNFRSF12A) have been identified as members of the TNF and TNF receptor (TNFR) family of cytokines, respectively [1]. A second scavenger receptor for TWEAK, CD163, has also been identified in recent years [3]. There is growing evidence of direct TWEAK/Fn14/CD163 involvement in several autoinflammatory pathologies across several tissue and cell types; however, the exact impact of TWEAK/Fn14/CD163 on disease development remains poorly understood. Recently, new functions for the TWEAK/Fn14/CD163 axis have become apparent, including the control of synaptic transmission and formation of atherosclerotic plaques [4, 5]. Here, we review the latest understanding of TWEAK/Fn14/CD163 as one of the chief regulators in immune signalling and its cell-specific role in metabolic disease development and progression.

## Structure of tweak and its receptors

**Fig. 1:**
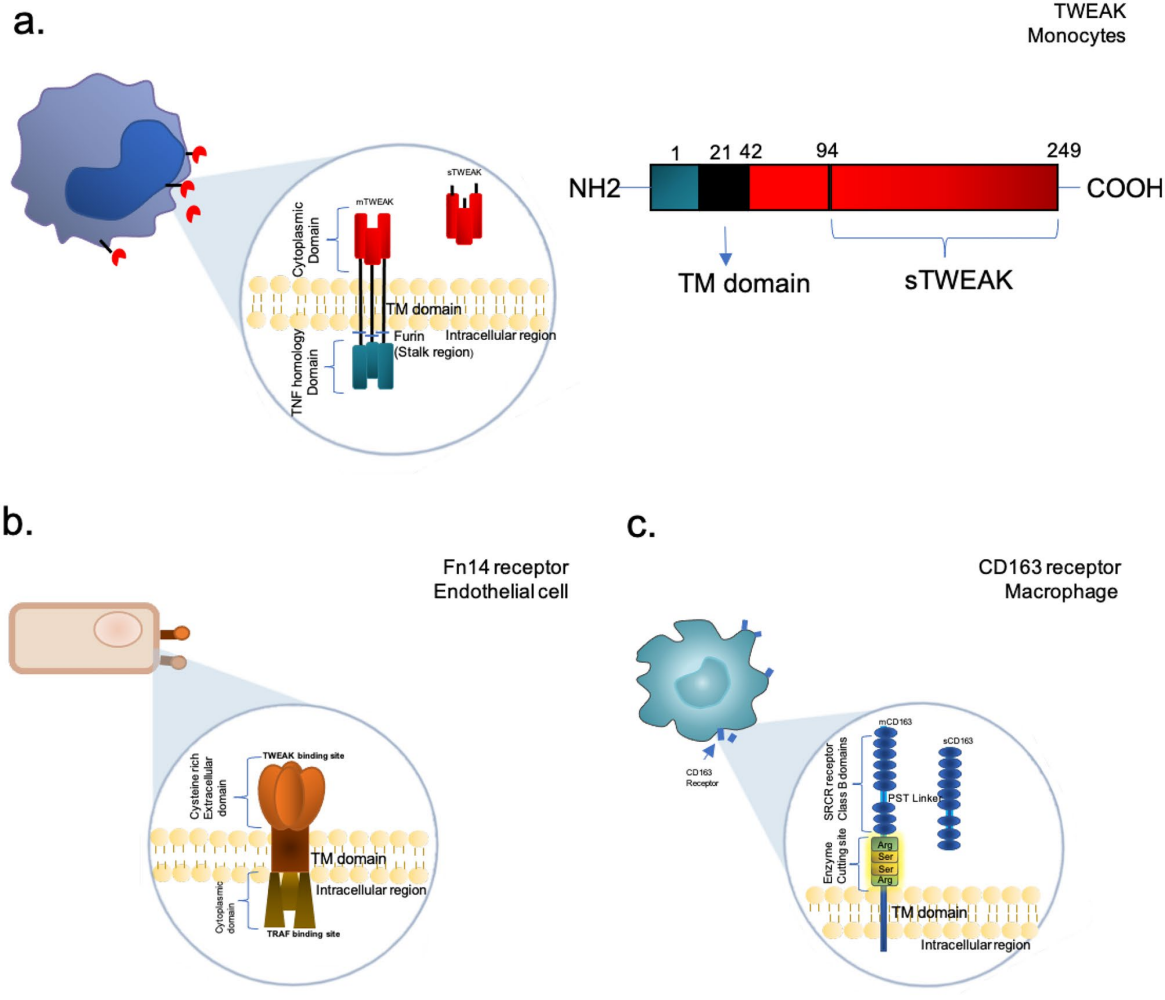
Structure of TWEAK and receptors. A Schematic representation of (**a**) membrane bound and soluble TWEAK produced and secreted by numerous cells type, mostly notably by monocytes, (**b**) the membrane Fn14 receptor expressed by several cells including endothelial cells and only detectable in large quantities in the event of cellular stress or injury, and (**c**) the CD163 receptor expressed exclusively on macrophages. The two receptors and TWEAK all contain transmembrane, intracellular and extracellular domains with CD163 and TWEAK existing in soluble forms. Localisation of the cellular TWEAK, CD163 and Fn14 axis interaction leads to different biological responses

In 1997, TWEAK was first identified as a novel, highly conserved and pro-apoptotic TNF-like protein in interferon gamma (IFNg) treated human HT-29 colon carcinoma cells [1, 6]. TWEAK exists in two forms—as a full-length membrane bound (mTWEAK) protein consisting of 246 amino acids, or as a 156-amino. acid soluble protein (sTWEAK) generated by furin proteolysis of TWEAK (Fig. 1a) [1, 7]. TWEAK has an intercellular N-terminal domain with a potential protein kinase C phosphorylation site, a transmembrane domain and an extracellular TNF homology domain (THD) located on the C-terminus [8]. TWEAK is also the only member of the TNF family which can bind the cognate Fn14 receptor – the smallest member of TNF receptor superfamily – and trigger signalling which can lead to growth and proliferation, angiogenesis, and in an inflammatory scenario, stimulation of apoptosis [9].

The extracellular ligand-binding region of Fn14 is composed of 53 amino acid residues and forms a single, cysteine-rich domain (CRD) similar to that of the fourth CRD of TNF receptor 1, which is a ubiquitous membrane receptor that binds TNFa (Fig. 1b) [8, 10]. The promoter region has multiple nuclear factor-kappaB (NF-κB) binding sites, enabling positive feedback regulation between Fn14 and NF-κB [11]. Protein modelling has revealed that intracellular signalling is only triggered after TWEAK binds to the receptor and enables trimerization of the TWEAK/Fn14 complex, which is enhanced by complementary binding interactions caused by opposing charge and hydrophobic residues [12]. Trimerization leads to higher order receptor clustering, activation of signalling and recruitment of intracellular adaptor proteins into the vicinity of the receptor [13]. Fn14 expression is normally low in healthy tissues and cells, but becomes upregulated in the event of injury, oxidative stress and inflammation [14, 15]. Numerous stimuli including endothelial growth factor, IFNγ, IL1β, thrombin and angiotensin can stimulate Fn14 expression [1]. Bacterial LPS also induces expression of Fn14 in SIEC02 cells in a TNFα-dependent manner [16]. However, TWEAK can suppress LPS-induced Fn14 expression in cells and reduce proinflammatory cytokine production [12]. Interestingly, when upper threshold levels of Fn14 expression are reached, TWEAK is no longer required to bind and initiate signalling and Fn14 can behave in a ligand-independent manner, activating the NF-κB pathway unilaterally [17].

The type I transmembrane scavenger receptor CD163 has been proposed as a scavenger receptor for TWEAK [3]. CD163 is expressed exclusively on the cells of the monocytic–macrophage linage and has been identified as the secondary, decoy receptor for TWEAK [3]. CD163 is a 1048 amino acid long membrane protein with a cytoplasmic tail, a single transmembrane section, and nine cysteine-rich scavenger receptor class B domains (Fig. 1c) [18]. It indirectly contributes to the anti-inflammatory response and can exist in various isoforms. Elevated plasma CD163 expression in macrophages is associated with increased inflammatory response sites [19]. CD163 binds to and internalises the available pool of sTWEAK which leads to regulation of TWEAK-induced activation of canonical NF-κB and Notch signalling needed for myogenic progenitor cell proliferation [20, 21]. There is growing evidence that differences in the concentrations of available mTWEAK and sTWEAK may have an impact on disease progression and outcome [22]. Patients with coronary artery disease, heart failure, chronic kidney disease and diabetes mellitus have been reported to have low levels of sTWEAK and increased Fn14 expression [22, 23]. However, the role of CD163 in this context has not been well investigated and the downstream effect of TWEAK/CD163 interaction is also unclear.

The biological effects of TWEAK appear to be cell and concentration dependent [24]. TWEAK is suggested to have a beneficial role in the acute, pathological environment by encouraging muscle repair. However, little is known about how TWEAK itself is regulated and which TWEAK regulated downstream pathways lead to repair and regeneration and which environments favour opposing effects.

## Tweak regulation of signalling pathways

### Canonical and non-canonical NF-κB pathways

The majority of the currently known functions of TWEAK are connected to expression of TWEAK-induced proinflammatory cytokines, chemokines, and cell adhesion molecules, predominantly through the activation of the NF-κB pathway, which is a highly regulated proinflammatory transcription factor that controls the expression of over 400 genes [25, 26]. TWEAK appears to be involved in the regulation of a multitude of genes; in vascular smooth muscle cells (VSMC) alone, it has been shown to upregulate 1611 and down regulate 1091 genes [27]. There is discussion around which pathways are influenced and regulated by TWEAK and what genes are affected; however, NF-κB pathway activation through Fn14 receptor signalling is the one in which the role of TWEAK is best understood. CD163 binding to sTWEAK has been shown to regulate the NF-κB pathway to some extent [21].

Binding of TWEAK to Fn14 recruits cellular inhibitor of apoptosis (cIAP1), TRAF2, TRAF5, and/or TRAF6 into a complex to activate transforming growth factor B-activated kinase 1 (TAK1), NF-κB -inducing kinase (NIK), and mitogen activated protein kinase kinases (MKK) [28]. Activation of TGF-B-activated kinase-1 TAK1 stimulates IB kinase (IKKB) leading to the activation of the early canonical NF-κB pathway characterised by nuclear translocation of RelA [28, 29]. NIK phosphorylates and activates IKKB leading to prolonged, but slower, activation of the non-canonical NF-κB pathway symbolised by increased DNA-binding activity of the p52/RelB NF-κB complex [23]. The non-canonical NF-κB pathway regulates immunity and disease development which is consistent with the ability of TWEAK to reduce innate response and its transition to adaptive TH1 immunity by curbing production of IFNγ and IL12 [4, 30].

The non-canonical NF-κB pathway is activated by a significantly smaller number of TNFR superfamily members—Fn14, TNFR2, BAFFR, CD40, LTBR, and RANK [28]. Many of these receptors can also activate the canonical NF-κB pathway and thus, intermediatory functional cooperation between these two pathways is required [28]. Interestingly, regardless of whichever NF-κB pathway is activated, the observed apoptotic behaviour of TWEAK is mediated through secreted TNFα, which via the TNFα-TNFR1 receptor complex, activates downstream RIPK1-FADD-Caspase-8 complex [31]. It was later proven that downregulation of otubain 1 (OTUB1) is needed to observe the TNFα mediation of apoptosis, as it enhances TWEAK or IAP antagonist‐stimulated c‐IAP1 degradation and decreases the level of apoptotic sensitivity [32].

### ERK/MAPK, EGFR and AP-1 signalling pathways

In addition to activating canonical and non-canonical NF-κB, TWEAK induces MAPK and activator protein-1 (AP-1) signalling pathways [28]. The activation of these pathways is attained through several different MKKs which activate Jun N-terminal kinase 1 (JNK1) and p38 MAPK. These recruit transcription factors including transcription factor AP-1, which regulates expression of genes involved in TWEAK-regulated responses [28].

TWEAK- controlled pathway activation appears to be cell-type specific and context-dependent [33]. TWEAK has a proinflammatory effect in adipocytes; however, this is mediated by NF-κB, and ERK pathways rather than JNK signalling [33]. TWEAK also increases proliferation in renal cells through activating the mitogen-activated protein kinases ERK and p38, the phosphatidyl-inositol 3-kinase (PI3K)/Akt pathway and NF-κB [34]. Anti-TWEAK monoclonal antibodies inhibit this proliferation through reduction in the expression of MAPK and NF-κB signalling as well as reducing AKT and p38 levels in autosomal dominant polycystic kidney disease preclinical models [35, 36].

Research data presents EGFR transactivation as a novel pathway for TWEAK–Fn14‐induced inflammation in kidneys [37]. Silencing of the *TNFRSF12A* gene stops TWEAK‐induced EGFR phosphorylation and due to a lack of tyrosine kinase activity within the Fn14 receptor, the EGFR pathway must be activated by intracellular mechanisms [37]. The same study indicates that binding of TWEAK to Fn14 activates ADAM17, a membrane‐anchored disintegrin and metalloproteinase important in kidneys, triggering secretion of HB‐EGF and TGFa that in turn, transactivates EGFR [30]. Inhibition of EGFR, ERK or ADAM17 *in vitro* effectively inhibits TWEAK-induced production of pro-inflammatory cytokines suggesting a role for TWEAK in the ADAM17–EGFR–ERK pathway regulation of pro‐inflammatory factor expression in a renal environment [37].

TWEAK, through mitogen activated protein kinase ERK and AKT signalling pathways, has been shown to impact cyclins (cyclinD1) and cyclin-dependent kinases (CDK4, CDK6) expression at both the protein and mRNA level and to decrease the expression of cyclin-dependent kinase inhibitors (p15lNK4B) in VSMCs, which is significant for regulation of proliferation [27]. Interestingly, inhibition of ERK1/2 activation by a MAPK kinase inhibitor has no effect on the TWEAK pro-calcific properties in VSMCs observed in chronic kidney disease, type 2 diabetes, and aging [38]. In the context of skeletal muscle, TWEAK has been shown to alter matrix metalloproteinase 9 (MMP-9) production through the activation of ERK1/2, JNK1, NF-κB -inducing kinase and p38 mitogen-activated protein kinase pathways [39]. The TWEAK induced increase in MMP-9 expression in myotubes was only impeded by the inhibition of p38 MAPK [39].

### Notch signalling

Notch signalling is a highly conserved signalling pathway almost universally present in every animal [40]. The activity of the receptor is highly regulated on all levels even at the post translational stage where numerous ubiquitin ligases and proteins, such as Numb are recruited to promote neural differentiation [40]. Notch signalling plays a significant role in embryonic development, determining the fate of T cell lineage from their lymphoid precursor [41]. Notch signalling is detected in the early stages of pancreatic development, where it directs the recruitment of endocrine cells from their progenitor cells, but many studies also indicate that its function extends to exocrine development [42]. Notch signalling has been implicated in several human pathologies of cardiovascular origin as well as in numerous cancers [43–46].

TWEAK has been shown to also activate Notch signalling through TWEAK-mediated activation of the canonical NF-κB pathway [21]. Interestingly the same study follows up with the effect CD163 has on TWEAK function, which is a relatively new area of exploration with the majority of studies focusing exclusively on Fn14 as the main modulator. CD163 has been shown to scavenge and inactivate sTWEAK which leads to poor outcomes in ischemic mice [21]. TWEAK was effectively prevented from mediating tissue damage repair at and beyond the site of injury and the duration of TWEAK-induced activation of canonical NF-κB/Notch signalling was shortened, which limited the differentiation of the progenitor cells [21]. CD163 deficient ischemic mice on the other had increased levels of TWEAK and enhanced notch signalling which aided in repair.

## Regulation of tweak expression and function

TWEAK expression and function appears to be controlled at multiple levels although regulation is poorly understood. TWEAK is similar to TNFα in that it is able to direct several biological responses [1, 47, 48]. During steady state, TWEAK unlike TNFα, can be detected at higher expression levels and is expressed in several more tissues which include heart, brain, kidneys, and mononuclear blood cells [23, 49]. *TWEAK* mRNA, by comparison to other TNF superfamily members is also more stable, enabling longer and more stable signalling times [49]. TWEAK can exist as a part of hybrid transcript consisting of *TWEAK* and *APRIL* (TNFSF13) mRNAs [50]. The encoded TWE-PRIL protein is made up of TWEAK cytoplasmic and transmembrane domains attached to the APRIL C-terminal domain [50]. *TWE-PRIL* mRNA is expressed and translated in human primary T cells, monocytes, and endogenous TWE-PRIL protein was detected in primary human T lymphocytes and monocytic cell lines [50]. It has been proposed, yet still to be confirmed, that the hybrid mRNA is not a by-product and in fact its expression is tightly regulated by an unidentified factor [50]. The same study has shown that TWE-PRIL is a membrane-bound, biologically active protein which has no effect on apoptosis but enhances cell division and proliferation [50].

The expression of *TWEAK* mRNA is downregulated after treatment with LPS in murine peritoneal macrophages, but the opposite is observed in human THP‑1 monocytic cells [1, 49]. TWEAK expression is quickly increased on monocytes when stimulated with IFNγ but not with IFNα [51]. TRAF3IP2 mediates TWEAK autoregulation and TWEAK-induced p38 MAPK, NF-κB and AP-1 activation [48]. c‐IAP proteins, which are critical E3 ligases, are also essential for the proper functioning of TWEAK [52]. In their absence TWEAK signaling and gene expression are greatly diminished [52]. OTUB1 works by inhibiting cytokine gene transcription within the immune system and regulates c‐IAP1 via K48‐linked polyubiquitination [53]. When OTUB1 is downregulated, it leads to much faster degradation of c‐IAP1 and as a consequence, weaker TWEAK signaling [52, 53]. Interestingly, only canonical NF‐κB and MAPK signaling are affected by the downregulation of OTUB1, which temporarily (as the non-canonical pathway is still functional) lowers the volume of produced cytokines including TNFα [32, 52]. TNFα is one of the most upregulated cytokines in these pathways, which on its own has no impact on c‐IAP1 K48‐specific ubiquitination or c‐IAP1 degradation [32]. In the absence of OTUB1 TNFα does however induce further TWEAK or IAP antagonist‐stimulated c‐IAP1 degradation and increases apoptosis [32, 52].

The list of downstream regulatory molecules which inhibit or activate TWEAK is unknown. However, it appears that TWEAK itself can act as a molecular switch dependent on the presence of angiogenic cytokines to act as a potential proangiogenic or antiangiogenic agent [46]. This is consistent with previous reports showing that TNF superfamily members can display seemingly dichotomous behaviors dependent on cellular conditions. During the early stages of acute inflammation, those cytokines and their receptors aid in the maintenance of homeostasis and encourage repair, whereas the opposing behavior of apoptosis and damage is observed during advanced chronic disease and inflammation [54]. Post-translationally, TWEAK protein function has been connected to induction of angiogenesis, cell growth and production of inflammatory cytokines [55]. It has been described as a weak inducer of apoptosis; however, depending on the cell type, it can also stimulate proliferation (for instance in liver cells and osteoblasts) [56]. This mirrors various mRNA expression responses to the same cytokines depending on the cell type. Little is known about how protein function is improved or maintained post-translationally. NFAT1-LCN2 can effectively regulate and stabilise TWEAK at the protein level [57]. NFAT1 induces Lipocalin 2 (LCN2) mRNA and protein expression by binding to specific sites in the *LCN2* gene promoter region and depending on the LCN2 expression, TWEAK displays either pro-tumorigenic or anti-tumorigenic behaviour [57]. NFAT1 and LCN2 are speculated to participate in the regulation of an unknown TWEAK receptor as LCN2 is necessary for TWEAK to promote its pro-invasive effect; in its absence, TWEAK can only signal by Fn14 which leads to anti-tumorigenic behaviour [57]. Poveda et al*.* have shown that TWEAK-induced NF-κB expression is regulated by Bcl3 and its over expression stops TWEAK-induced inflammatory and lethal responses in cultured tubular cells [23]. Bcl3 is a NF-κB regulatory protein of the IκB family, which by ubiquitination of P50 and P52, inhibits DNA binding and gene transcription [23]. P50 and P52 are important members of the non-canonical NF-κB through which TWEAK primarily signals [30].

Cell extrinsic factors such as hyperglycaemia or reactive oxygen species play an important role in the expression and stability of many of the proteins of the TNF superfamily [47]. Very little information is available on how and if these factors affect the expression of TWEAK directly. A study by Padrão et al. has demonstrated that regular exercise encouraged upregulation of PGC‐1a and oxidative phosphorylation complexes and simultaneously prevented TWEAK from displaying its pro-invasive behaviour in the context of cancer [58]. Obesity and insulin resistance have also been identified as potential factors leading to lower plasma sTWEAK concentrations, which in the case of cardiovascular disease appear to be detrimental [59]. Further work is required to elucidate how extrinsic factors affect TWEAK expression and signalling.

## Tweak and diabetes

One of the main problems associated with the lack of a permanent ‘cure’ for diabetes versus long term treatment as a way of managing the disease, is the fact that once damaged, beta cells have a limited ability to proliferate and expand to restore insulin production [60]. In type 1 diabetes (T1D), T cell mediated destruction of beta cells is accompanied by trans- and de-differentiation of beta cells to glucagon-producing alpha cells [61]. NGN3 is an important regulatory transcription factor involved in neurogenesis; it is essential for endocrine cell fate specification in multipotent intestinal progenitor cells [62]. NGN3 positive cells obtained from exocrine cultures have characteristics typical of endocrine progenitor cells during early-stage development [63].

**Fig. 2:**
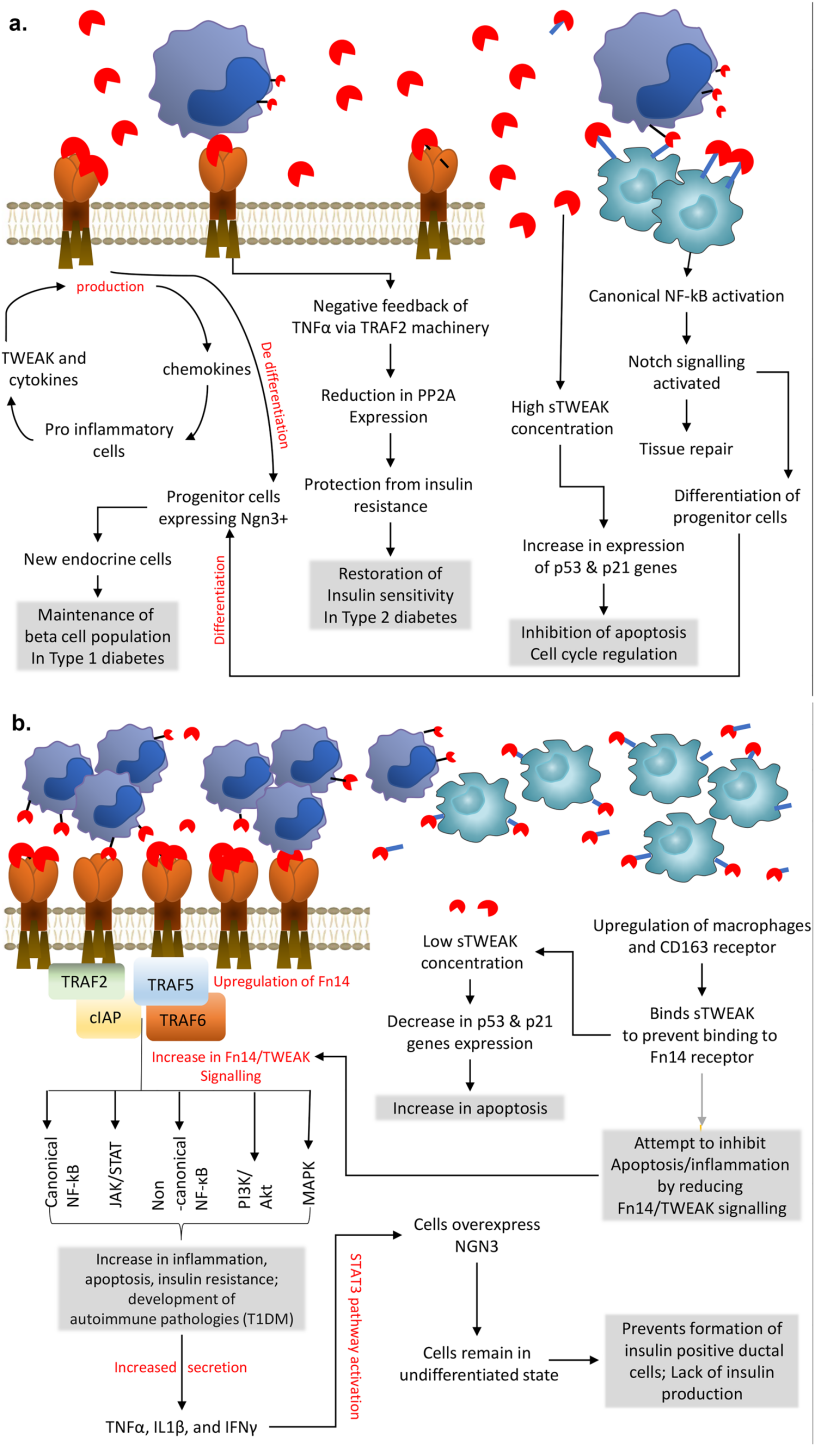
Proposed mechanisms of the TWEAK/Fn14/CD163 axis in the context of diabetes. In normal homeostatic conditions (**a**) TWEAK/Fn14/CD163 axis of interaction in pancreatic cells, adipose and muscle cells shows a protective role which allows for generation of new endocrine cells, cellular repair, protection from insulin resistance, regulation of cell cycle and apoptosis. In inflammatory conditions observed during chronic hyperglycaemia (**b**) there is an observed increase in infiltration by TWEAK expressing macrophages, increase in CD163 expressing monocytes and increase in Fn14 receptor expression. Detected plasma sTWEAK concentration is lower in persons with diabetes. It is proposed that CD163 scavenges sTWEAK to inhibit its interaction with Fn14 and stop downstream interactions. These processes lead to apoptosis, reduction in insulin production and sensitivity. Shaded boxes indicate the final observed effect of TWEAK signalling

TWEAK-Fn14 interaction in healthy adult mice induces proliferation of ductal cells which are part of the pancreatic epithelial lining involved in the transfer of exocrine enzymes from acinar cells into the duodenum [64, 65]. It also induces transient NGN3 expression in the healthy normal adult mouse pancreas and those NGN3 positive cells do not express islet hormones [65]. Similar observations can be made in the early stages of pancreatic development in embryos [65]. TWEAK-Fn14 interaction could potentially facilitate the production of new endocrine beta cells by de-differentiating ductal cells into progenitor cells expressing NGN3 (Fig. 2a) [65]. Those cells could through normal cellular machinery undergo neogenesis, become new beta cells and restore insulin production [65]. This study has not however, addressed the effect of hyperglycaemia or inflammation on the TWEAK-NGN3 relationship and has not described the role of NGN3 at each stage of cellular maturation. Proinflammatory cytokines (TNFα, IL1β, and IFNγ), which are upregulated in the diabetic environment also upregulate NGN3 in human ductal cells via STAT3 signalling and this upregulation is persistent [66]. In this case, the prolonged NGN3 stimulation by cytokines in a chronic inflammatory environment such as diabetes appears to retain cells in an undifferentiated condition and prevent the formation of insulin positive ductal cells [66].

Obesity drives type 2 diabetes (T2D) development [67, 68]. There is drastically less circulating sTWEAK in the plasma of people with T2D, yet, *TWEAK* gene expression in subcutaneous adipose tissue is not affected by obesity with *Fn14* expression being detected only in patients with a BMI > 38 kg/m^2^, [33, 69]. The adipose tissue plays an important in the maintenance of energetic homeostasis and can quickly respond to changes in caloric intake via adipocyte hypertrophy and hyperplasia [70]. The increase in adipose tissue observed during obesity has been shown to encourage several cell intrinsic behaviours such as adipocyte apoptosis, hypoxia, mechanical stress, abnormal secretion of chemo-attractants and consequential triggering of inflammatory responses [71]. Increased macrophage recruitment is observed alongside an increase in adipocyte death [72]. Macrophages make up to 40% of all adipose tissue cells in obese subjects [72]. TNFα, another member of the TNF superfamily, is significantly increased in the subcutaneous adipose tissue of morbidly obese people with and without T2D [69]. TNFα is a potent inducer of the NF-κB pathway, leading to insulin resistance and an increased inflammatory state, which are chronically observed in obese subjects [73]. Despite this, a reduction in circulating TNFα does not restore insulin sensitivity in people with T2D [74]. Obese adipose tissue is infiltrated by macrophages which are a source of mTWEAK and sTWEAK [33]. sTWEAK is a negative feedback regulator of TNFα signalling, predominantly through TRAF2 [33]. sTWEAK has been shown to protect from insulin resistance by down regulating the TNFα induced increase in the activity of Protein phosphatase 2 (PP2A) (Fig. 2a) [33]. Nutritional therapies and bariatric surgery are at present the most common and effective methods of fighting obesity [75]. Currently, there are no studies showing implementation of sTWEAK as an adjunct therapy to nutritional changes. However, in 69% of bariatric surgery patients, there was an increase in previously downregulated sTWEAK levels thereby hindering TNFα intracellular signalling events and lowering inflammation [76]. This suggests that TWEAK could act as a post-bariatric surgery anti-inflammatory therapy to aid in recovery.

In the study by Simon-Muela et al. gestational diabetes mellitus has also been connected with lower levels of circulating sTWEAK [77]. Yet, CD163 or CD163/sTWEAK were negatively associated with HOMA‐IR [77]. Interestingly, downregulation of TWEAK in uterine natural killer cells (uNK) of pregnant rats (which are the most common lymphocytes present during the early stages of gestation) and subsequent exposure to LPS leads to changes in cytotoxicity of those cells which encourages foetal rejection [16]. This suggests the role of TWEAK in maintaining homeostasis is needed for successful pregnancy. Lower levels of sTWEAK were also associated with depression in people with T1D compared to those with T1D without depression [78]. There is no clear mechanism available which would describe how sTWEAK levels are depleted. Some studies have suggested that the scavenger receptor CD163 is upregulated in the presence of pathologies characterised by chronic inflammation such as atherosclerosis and T2D allowing it to neutralise the biological activity of sTWEAK [79, 80]. The topic remains controversial as contradictory reports have been put forward questioning if CD163 concentration is a meaningful measure of metabolic syndrome and low grade inflammation [20, 79–81].

The controversy surrounding TWEAK and its receptors is further deepened by studies showing that TWEAK-transgenic mice have significantly higher body weight/fat mass in addition to increased insulin resistance and that TWEAK may promote poor disease outcomes in metabolic pathologies (Fig. 2b) [82]. Similar observations have been made for nephropathy/diabetic nephropathy where TWEAK administration exacerbated renal damage in ApoE-knockout mice [82]. However, patients with diabetes undergoing haemodialysis showed low levels of TWEAK [83, 84]. The studies propose that TWEAK binds to Fn14 with increased magnitude, as the expression of the receptor is increased, and remainder of TWEAK is being scavenged by CD163 effectively leading to lower detection levels in plasma [83, 84].

The use of resveratrol decreases CD163 expression which has been shown to increase levels of sTWEAK in people with T2D [85]. Resveratrol also increases expression of SIRT1 and 5′ AMP‐activated protein kinase (AMPK), which together inhibit NF‐κB and therefore, further increase sTWEAK concentration [46, 85]. The increase in sTWEAK was connected to increased expression of p53 and p21 genes required for inhibition of apoptosis, cell cycle regulation and tumour suppression [46, 85].

## Tweak and cardiovascular disease

Over 75% of diabetes-related mortality is due to cardiovascular disease [86]. Cardiovascular diseases are associated with atherosclerosis which is an inflammatory disease recognised by an abnormal accumulation of macrophages in the walls of the blood vessels [87]. Macrophages can display pro- or anti-inflammatory effects dependent on their polarisation state, which is a significant factor in the development of atherosclerotic plaque [88]. Similar to other inflammatory diseases, the detectable sTWEAK concentration is significantly lower in patients with carotid atherosclerosis, coronary and peripheral artery disease, atherosclerosis caused by T2D, and end stage renal disease [83]. CD163 levels are increased in those patients, however. CD163 has been recognised as an atherosclerotic plaque modulator due to its anti-inflammatory and anti-atherogenic abilities [89, 90]. It is, however, worth noting that numerous diseases such as acute kidney injury, autosomal dominant polycystic kidney disease and rheumatoid arthritis display upregulation of both TWEAK and Fn14 [36, 91–93]

There is a negative correlation between sTWEAK and IL6, and together with the low-grade inflammation score, which in turn is associated with arterial stiffness, is an early precursor to atherosclerosis [59]. In some studies, CD163 concentration has also been associated with inflammatory markers [20]; however, Llaurado et al*.,* did not observe this in their cohort, with CD163 only displaying a modest association with low sTNFαR2 exclusively in males [59]. Despite this, a CD163 deficiency appears to have a negative impact on the atherosclerotic outcome in mice models. ApoE/CD163 double-deficient mice displayed bigger plaques, greater instability in their atherosclerotic plaques, elevated lipid-macrophage concentration, and higher expression of pro-inflammatory cytokines [94]. The same study demonstrated that CD163 deficient M2 macrophages resulted in greater foam cell formation via the upregulation of CD36. Direct blockage of the TWEAK/Fn14 pathway significantly decreases the uptake of oxidised low-density lipoprotein by macrophages, which reduced foam cell formation, but this is not regulated by changes in CD36 expression [95]. Interestingly foam cells themselves express Fn14, yet TWEAK/Fn14 interactions on their own do not control apoptosis of macrophage foam cells *in vitro.* Furthermore, Fn14-Fc treatments *in vivo*, do not affect apoptotic rates in atherosclerotic plaques [95]. Increases in apoptosis are only observed when the cells are stimulated with a cytomix of TWEAK and TNFα [95].

**Fig. 3:**
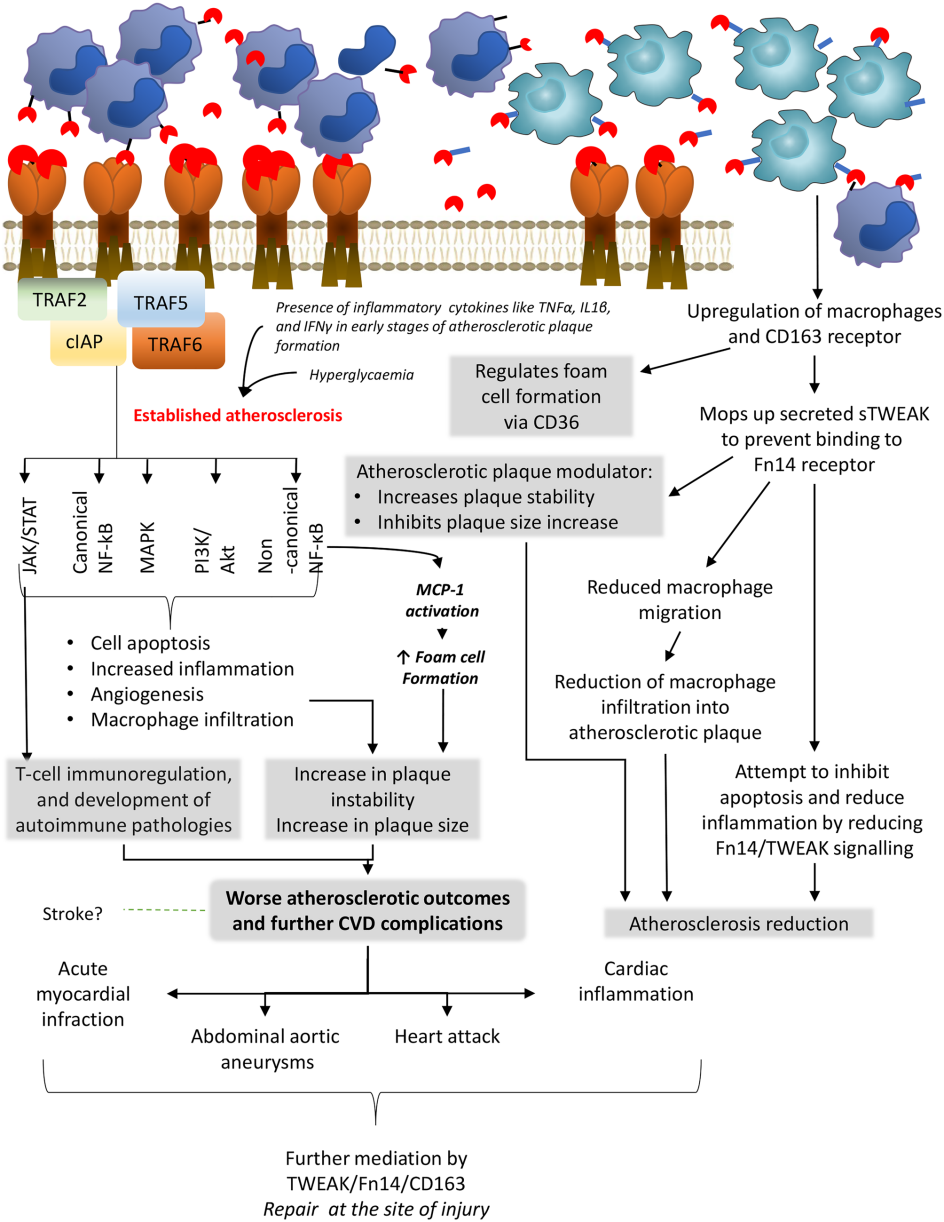
Proposed mechanism of the TWEAK/Fn14/CD163 axis in the context of cardiovascular disease. TWEAK/Fn14/CD163 have been implicated in the development of CVD with a large focus on atherosclerotic plaque formation and development. Similar to other inflammatory pathologies, in atherosclerosis, there is increased infiltration by TWEAK expressing macrophages, increased CD163 expressing monocytes and increased Fn14 receptor expression. The concentration of sTWEAK is also reduced. It is proposed that CD163 binds to TWEAK, which stops macrophage migration and further infiltration. It also helps in maintenance of plaque stability and size although the downstream pathway responsible for this is not known. Initial plaque development is independent of TWEAK, but through the interaction with Fn14 and presence of other cytokines, TWEAK impacts established plaques leading to an increase in plaque size and instability, which is largely driven by foam cell formation and angiogenesis. Atherosclerosis can lead to several cardiovascular complications such as myocardial infraction and heart attacks. There is some evidence that TWEAK/Fn14/CD163 is involved at the sites of the acute injury caused by cardiovascular (CVD) and is largely used for repair and not further damage. Shaded boxes indicate the final observed effect of TWEAK signalling

Addition of recombinant CD163 diminishes the migration of macrophages induced by supernatants from VSMCs stimulated with TWEAK [94]. This falls in line with previous studies where blocking of TWEAK encouraged cell migration *in vitro* yet at the same time alleviated the number of cells infiltrating into diseased tissue. This suggests that TWEAK regulates chemokine gradients, adhesion and cell migration dependent on the environmental conditions [95, 96]; the mechanism behind this dual action is not known. Gutierrez-Muñoz et al. also state that CD163 expressing macrophages exert a protective role by blocking TWEAK’s effect on atherosclerosis development and progression (Fig. 3) [94]. There is evidence however, that soluble Fn14, which is upregulated during inflammation, can inhibit TWEAK/CD163 interaction [3, 97]. Binding of CD163 to TWEAK does not denature the protein or stop its activity, but instead activates different downstream pathways like Notch signalling needed for repair [21]. There is little information available on the behaviour of TWEAK and its mechanism of action in the context of atherosclerotic inflammation and metabolic disease in various cells; however, it could be speculated that increases in CD163 act to counteract increased Fn14 expression and competitively bind to TWEAK to simultaneously encourage potential beneficial CD163-TWEAK interactions and negate negative Fn14-TWEAK interactions. Studies suggest that TWEAK has no effect in plaque initiation but instead exerts its effects in the later stages of already developed atherosclerosis [27, 95]. This could be influenced by the increased pool of other cytokines, such as TNFα. TNFα is actively involved in the progression of atherosclerosis and influences TWEAK/Fn14/CD163 activity, which through regulatory cascades, leads to worse atherosclerotic outcomes [98]. In fact, Fernández-Laso et al. who focused on the direct effect of TWEAK on atherosclerosis in diabetic mice (rather than that of the receptors), proposes that the reduction in the presence of inflammatory cells (mostly macrophages) in lesions of TWEAK deficient diabetic mice is due to decreased activation of JAK/STAT, NF-κB, and the expression of proinflammatory cytokines [55]. This was also the first study to show that hyperglycaemia induces TWEAK to display its pro-atherosclerotic behaviour by partially inhibiting STAT1 and consequently upregulating the expression of proinflammatory target genes including CCL5, CXCL10 and ICAM-1. TWEAK functioning through STAT1 is an interesting discovery. There is evidence that STAT1 plays a significant role in beta cell death, T-cell immunoregulation, and development of autoimmune pathologies such as T1D [99]. The mechanism by which STAT1 gain of function encourages autoimmunity is not known but it has been put forward that faulty lymphocyte activation and signalling are the key culprits [99]. In fact, use of JAK inhibitors to inhibit STAT1 show improvement in NK cell function, and dysregulated T-cell polarisation in patients with STAT1 gain of function mutations [100].

The TWEAK/Fn14 pathway appears to be involved in cardiac inflammation in non-ischemic stress conditions (Fig. 3). Unudurthi et al., have demonstrated that it is the Fn14 receptor which mediates pressure overload-induced heart failure, macrophage infiltration in hearts exposed to this kind of pressure, and that its inhibition can effectively reduce the pathological remodelling and prevent further cardiovascular complications to some extent [101]. This study has also shown that increases in MCP-1 secretion in cardiac fibroblasts is mediated by the TWEAK-Fn14 pathway partially through engagement of the non-canonical NF-κB pathway [101]. MCP-1 through its chemotactic inducing abilities encourages passage of monocytes from the lumen to subendothelial space where they convert into foam cells, which eventually leads to atherosclerotic plaque formation [102]. Interestingly, increases in the available circulating plasma MCP-1 have also been connected with increased long-term risk of stroke [103]. Recent studies have likewise shown that secreted MCP-1 regulates the angiogenic effect of tissue factor by recruiting smooth muscle cells toward endothelial cells and enables the maturation of newly formed micro vessels [104].

Atherosclerosis can also lead to acute myocardial infraction (AMI) which is one of the leading causes for death worldwide [105]. Similar to atherosclerosis, plasma sTWEAK expression is significantly higher in patients who suffered from AMI. However, in this instance, TWEAK encourages beneficial endothelial progenitor cell (EPC) vasculogenic properties to relieve acute myocardial infarction via the Fn14‑NF‑κB signalling pathway and additionally increases the migration of EPC to the site of injury [106]. Although not directly related to cardiovascular disease (CVD), it is worth mentioning that TWEAK/Fn14 activation has been shown to induce beneficial migration and cytokine production of both dermal microvascular endothelial cells and dermal fibroblasts in burn wounds [46]. What is interesting is that TWEAK mediates and increases the expression of α-SMA and palladin in dermal fibroblasts during repair [46]. Recent studies have made a connection between reduced levels of α-SMA and increased abdominal aortic aneurysms [107]. It is speculated that abdominal aortic aneurysms (although not often considered a ‘standard’ cardiovascular disease) are largely caused by atherosclerosis [108].

The expression of TWEAK/Fn14 in healthy human brain and their regulation in inflammatory and neurodegenerative diseases are yet to be characterised. There is, however, evidence that TWEAK/Fn14 plays a role in stroke development [109]; CD163 has not been well investigated in this context. There is a sharp upregulation of sTWEAK concentrations and Fn14 expression in ischemic stroke patients with the concentration of TWEAK returning to normal within 24 h after a stroke event [109, 110]. Light induced increases of Fn14 in the thalamocortical excitatory neurons and up-regulation of TWEAK in microglia is required for the removal of weak synapses and fortifying of the remainder, which is required for correct synapse maturation [111, 112]. The role of TWEAK/Fn14 outside of synapses is however, poorly understood. In the study by Nagy et al. it was demonstrated that inhibition of TWEAK/Fn14 is beneficial post ischemic stroke as it appears to limit synaptic degradation and increase basal synaptic transmission and plasticity in Fn14KD mice [5]. Other cytokines such as IL1β and TNFα can also be found at the site of injury post ischemic stroke [113]. Interestingly TWEAK cannot induce synaptic apoptosis on its own unless other cytokines such as TNFα and IFNγ are present [1, 5].

## Conclusions

The general theme of current research suggests that chronic inflammation is the main causative agent which causes TWEAK to behave erroneously and propagate inflammation-driven cellular degradation. Significantly more work is required to understand the role of the TWEAK/Fn14/CD163 axis alongside other inflammatory cytokines in the context of diabetes. Despite increases in research investigating TWEAK and its receptors, especially in the cardiovascular setting, a lot of questions remain unanswered. It is unknown if membrane bound TWEAK has a different effect on signalling and its ability to bind to neighbouring Fn14 receptors or even CD163. Furthermore, a deeper understanding on the preferential activation of pathways to induce certain cellular behaviours in different tissue types is required. In fact, there are very few studies investigating the effect of the TWEAK/Fn14/CD163 axis in brain, heart or pancreas, before the complexities of extrinsic cellular factors are included. TWEAK/Fn14/CD163 undoubtably has therapeutic potential, yet there is a need to establish what factors cause the switch in the function of TWEAK from protective to damaging.
